# Recent Advances in Reactive Ion Etching and Applications of High-Aspect-Ratio Microfabrication

**DOI:** 10.3390/mi12080991

**Published:** 2021-08-20

**Authors:** Michael Huff

**Affiliations:** Founder and Director of the MEMS and Nanotechnology Exchange, Corporation for National Research Initiatives, Reston, VA 20191, USA; mhuff@mems-exchange.org

**Keywords:** reactive ion etching, high-aspect ratio etching, inductively-coupled plasma etching, micromachining, MEMS, NEMS

## Abstract

This paper reviews the recent advances in reaction-ion etching (RIE) for application in high-aspect-ratio microfabrication. High-aspect-ratio etching of materials used in micro- and nanofabrication has become a very important enabling technology particularly for bulk micromachining applications, but increasingly also for mainstream integrated circuit technology such as three-dimensional multi-functional systems integration. The characteristics of traditional RIE allow for high levels of anisotropy compared to competing technologies, which is important in microsystems device fabrication for a number of reasons, primarily because it allows the resultant device dimensions to be more accurately and precisely controlled. This directly leads to a reduction in development costs as well as improved production yields. Nevertheless, traditional RIE was limited to moderate etch depths (e.g., a few microns). More recent developments in newer RIE methods and equipment have enabled considerably deeper etches and higher aspect ratios compared to traditional RIE methods and have revolutionized bulk micromachining technologies. The most widely known of these technologies is called the inductively-coupled plasma (ICP) deep reactive ion etching (DRIE) and this has become a mainstay for development and production of silicon-based micro- and nano-machined devices. This paper will review deep high-aspect-ratio reactive ion etching technologies for silicon, fused silica (quartz), glass, silicon carbide, compound semiconductors and piezoelectric materials.

## 1. Background

Prior to the advent of reactive ion etching, the etching of silicon semiconductor substrate materials using microfabrication methods was performed using either a wet chemical-based immersion or a conventional plasma dry etching process [1,2,3]. Both of these processes etch silicon using a chemical reaction. While these methods were able to provide high levels of mask selectivity, as defined by the etch rate of the material etched relative to the etch rate of the masking layer material), the etch rate proceeds nearly equally in all directions; that is, these techniques are mostly isotropic. Isotropic etching results in severe undercutting of the masking layer and makes it nearly impossible to accurately control the dimensions of the etched features. This problem becomes severe for etches into the substrate to any significant depth as needed for bulk micromachining, which is defined as the selective removal of substrate material in order to implement three-dimensional shapes and structures. Bulk micromachining is an essential fabrication method in many microsystems and micromachined devices, including Micro-Electro-Mechanical Systems (MEMS) and Nano-Electro-Mechanical Systems (NEMS) [4]. Although there is no defined depth required for an etch to be classified as bulk micromachining, in practice, the depth of the etching is at least 10 microns or more, and often is through a considerable portion of the thickness of the substrate (i.e., hundreds of microns).

Typically, any bulk micromachining process is performed by first depositing a masking material layer and then patterning it to expose areas on the surface of the substrate, followed by the substrate being etched in the exposed regions [4]. Since bulk micromachining fabricates microsystems devices directly from the material of the substrate, it affords specific benefits to microsystems devices since the substrate is composed of a high-quality single-crystal material (e.g., single-crystal silicon). This is in comparison to implementing microsystems devices from deposited thin-film materials layers using methods such as chemical vapor deposition (CVD) or physical vapor deposition (PVD) methods that result in material layers that are amorphous or polycrystalline and typically have inferior and process-dependent material properties (e.g., residual stresses and stress gradients). The superior mechanical and electrical material properties of single-crystal silicon substrate materials enable excellent device performance with known and predictable material properties [5]. This is extremely important for implementing many micromachined devices.

Bulk micromachining etching processes preferably have several attributes, including [4,6]: a high level of anisotropy; nearly vertical etch sidewalls that are as smooth as possible; excellent uniformity across the substrate and from substrate to substrate; the ability to implement high-aspect-ratio features; the ability to etch deep into the substrate material including etching completely through entire substrate thickness; good selectivity with conventional masking material layers such as photoresist, silicon dioxide, and silicon nitride; low cost; repeatable and reproducible performance; and low environmental impact.

Etching technologies for bulk micromachining have radically evolved over several decades that they have been practiced. Various methods have been demonstrated using a variety of chemical, physical, and combined chemical and physical processes. Purely chemical techniques involve a chemical reaction between a reactive species (usually in the form of either a liquid solution or gas) and the substrate material [7,8,9,10]. Purely physical etching techniques usually involve removal of substrate material using momentum transfer of energetic chemically inert particles [1,2]. The most notable example of this type of etch process is ion milling. The combination of chemical and physical techniques unites chemically reactive species with momentum transfer and thereby exhibits both selectivity and anisotropic behavior. RIE etching falls into that later category and therefore these types of etch processes tend to provide the best characteristics for obtaining the many of the desirable etching attributes for advanced microfabrication etching [1,2,3,11,12].

## 2. Dry Etching Techniques

**Figure 1:**
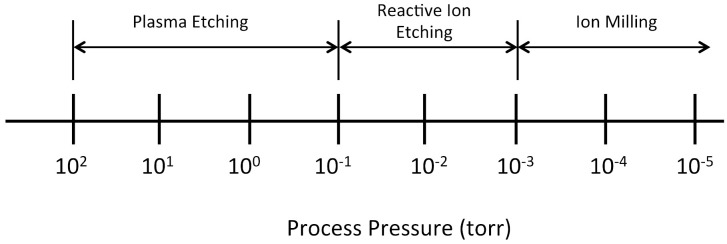
Spectrum of dry etching processes and their relationship to the process pressure [13].

The relative amount of chemical and physical mechanisms present in a given dry etch process varies depending on the type of plasma process employed [1,2,3]. In general, dry etching encompasses a spectrum of different types of technologies as shown in Figure 1, ranging from plasma etching, to reactive ion etching (RIE), to ion milling. An important differentiator among these different technologies is the process pressure used in the etch chamber during etching.

**Figure 2:**
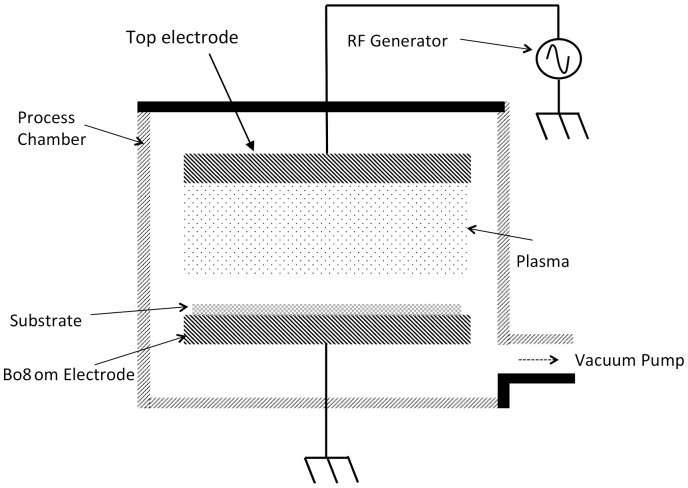
Illustration of a plasma etching system. The substrate is positioned on the bottom electrode that is electrically grounded and the top electrode is connected to a RF generator [13]. Inlet process gas lines are not shown.

Plasma etching is performed at comparatively higher process pressures (i.e., 10^−1^ to 10^2^ torr). The constituents of the plasma have relatively short mean free paths and experience multiple collisions before striking the substrate. Due to these collisions, the etching species impinge the substrate over a range of angles thereby making this form of dry etching more isotropic in nature. Consequently, plasma etching is mostly driven by the chemical reactions of the plasma species with the exposed material to be etched and not as a result of the physical effects. Like wet etching, plasma etching is not very directional, but tends to be material selective. Figure 2 illustrates a traditional plasma etcher configuration.

Advanced microsystems manufacturing requires the implementation of very small features and etching processes that are highly anisotropic are required [13]. As such, plasma etching is rarely used in microsystems manufacturing, except in very specialized applications such as resist stripping.

At the other end of the spectrum (Figure 1), the process pressures are 10^−3^ torr or lower and the mean free path of the species is significantly longer, typically longer than the reaction chamber. Given that the plasma species do not experience collisions, they are able to maintain higher energy levels and impart that kinetic energy into the surface atoms when they impinge onto the substrate. This form of dry etching is called ion milling and is purely based on the mechanical bombardment of the plasma ions striking the substrate surface; not on any chemical reaction. Ion milling is a very anisotropic etch process, with a minimal lateral etch rate. However, ion milling is non-selective. Consequently, it is not useful for deep etching (more than a few microns) into materials. Nevertheless, it is commonly used for etching materials that cannot be etching using other means.

At process pressures between the levels of 10^−1^ and 10^−3^ torr resides reactive ion etching (RIE). RIE combines the attributes of both plasma etching and ion milling. In RIE, the impacting plasma species impart significant kinetic energy into the atoms of the substrate surface to initiate the etching and the chemically reacting species react with the substrate atoms, which then desorb from the surface as reaction by-products. As a result, RIE is more material specific than ion milling, while at the same time provides good anisotropy of the etched features.

## 3. Specifics of Reactive Ion Etching (RIE)

**Figure 3:**
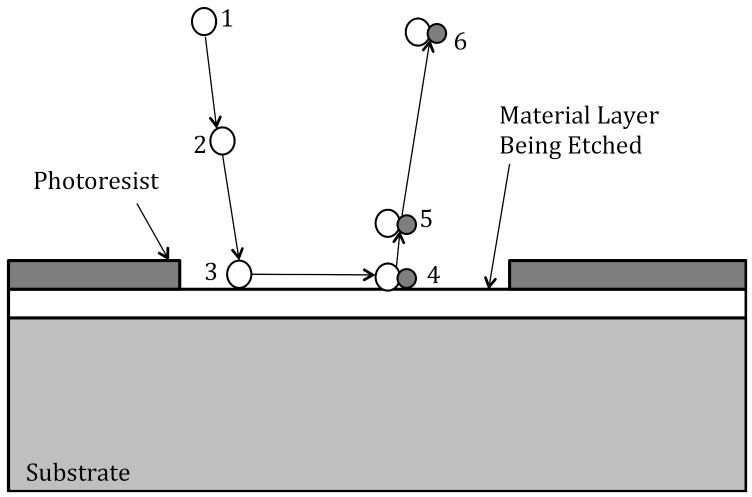
Illustration of the six steps involved in plasma etching [14]. Step 1: process gases are broken into chemically reactive species in plasma; Step 2: diffusion of reactive species to substrate surface; Step 3: absorption of reactive species onto material layer; Step 4: reaction between reactive species and material layer; Step 5: desorption of reaction by-products; Step 6: diffusion of by-products.

The RIE plasma etch process involve six steps and each step must occur for the etching to proceed (see Figure 3) [14]. First, process gas(es) introduced into the etch chamber and are broken down into chemically reactive species (i.e., radicals) by the plasma. Second, the reactive species diffuse to the substrate surface. Third, the reactive species are absorbed onto the substrate surface. Fourth, the chemically reactive species move about the surface and chemically react with the substrate. Fifth, the reaction products desorb from the substrate surface. Sixth, the reaction products are carried away out of the etch chamber. The etch rate is determined by the step having the lowest rate.

**Figure 4:**
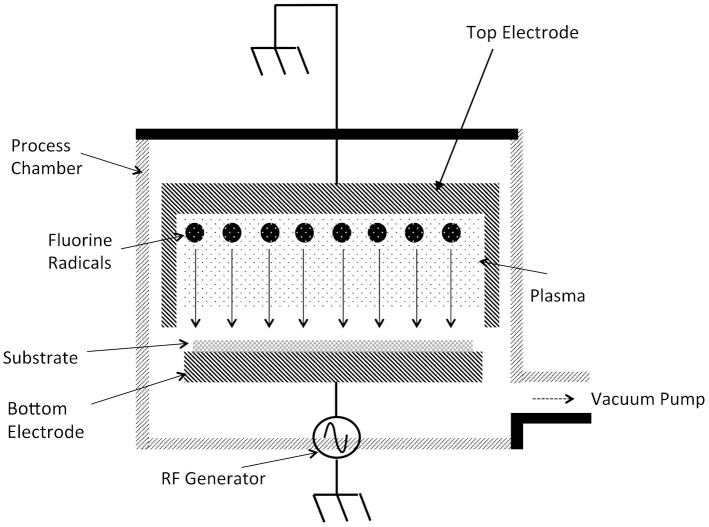
Illustration of a reactive ion etch (RIE) etching system [13]. Inlet process gas lines are not shown.

The equipment of a RIE etcher involves process chamber connected to a vacuum pump to achieve the proper operating pressures (see Figure 4). Process gases flow into the etch chamber in controlled levels using in-line mass-flow controllers (not shown in Figure 4). RIE etchers are typically single wafer etch tools since this provides for better uniformity. Most etchers in production use load-lock chambers to increase wafer throughputs. The substrate to be etched is placed on a chuck that also acts as an electrode. This electrode is electrically connected to an RF generator operating at a frequency of 13.56 MHz and up to a few hundred Watts of power. The chamber walls and top electrode are electrically grounded. The electromagnetic field applied to the process gases into the etch chamber result in the electrons being stripped from the gas molecules thereby initiating the plasma.

The electrical grounding of the chamber walls and top electrode that have relatively large areas and applying the electromagnetic field to the bottom electrode that has a much smaller area results in increased ion energies that impact onto the substrate [2,3,13]. As the electromagnetic field oscillates, electrons in the plasma impact both the chamber walls and substrate. The plasma ions on the other hand, are much more massive and therefore are not able to travel distances equivalent to that of the electrons. Electrons that impact the chamber walls are absorbed since these surfaces are electrically grounded. The electrons striking the substrate result in a large negative charge to be created since this surface is DC isolated. Simultaneously, the plasma sheath builds up a positive charge due to the higher concentration of positive ions compared to free electrons. As a result, a large voltage potential develops on the substrate electrode relative to the top electrode and chamber walls. The potential difference between the substrate electrode and the plasma sheath is usually a few hundred volts and results in the ions in the plasma to be attracted to and impact the substrate with high kinetic energies thereby imparting significant mechanical energy into the substrate surface.

RIE etching processes use gases that contain halogens, which are group VII elements, including, fluorine, chlorine, bromine, iodine, due to the fact that these elements are highly electronegative and reactive. When these halogens react with the material being etched, they form chemical compounds known as halides. The volatility of the resulting halide is an important parameter and for an etching process it is desirable that the halides have a high vapor pressure. Specifically, the different process gases that are employed in RIE of different material types include fluorine-based gases such as SF_6_ and CF_4_ for the etching of silicon and silicon carbide; fluorine-based gases such as C_3_F_8_ and C_2_F_6_ for the etching of silicon dioxide and silicon nitrides; chlorine-based gases such as BCl_3_ and CCl_4_/Cl_2_/BCl_3_ for the etching of aluminum, other metals, and compound semiconductors; oxygen-based gases such as O_2_ and CO_2_ for the etching of organics such as photoresist; other chemistries for other material types such as silicides and refractory metals.

The advantages of RIE etching include good depth uniformity; good mask selectivity; less chemical waste handling issues (compared to wet etching); relatively clean process; can provide high fidelity and dimensional control of the etched features; and amenable to automation for cassette-to-cassette high-wafer-throughput production. Because of these desirable attributes and others, RIE is widely used etch technology in IC manufacturing [2,3,13].

## 4. Reactive Ion Etching (RIE) Considerations

There are a number of important considerations for RIE etch processes [13,14,15]. The etch rate is defined as the depth of the etching per unit time. A higher etch rate is more attractive since it enables faster process times thereby increasing wafer throughput and lower production costs. Nevertheless, it is also important that the etch rate is controllable. RIE processes generally have etch rates in the range of hundred to thousands of Angstroms per minute. Importantly, the etching rate may not be constant as the depth of the etch continues, especially for high-aspect-ratio features. This is due to the constrained diffusion of the etchant gases from the plasma to reach the bottom of narrow trenches and the inability of the reaction by-products to escape. Therefore, it is common for high-aspect-ratio etches to exhibit decreasing etch rates as the depth of the etch increases.

Another important consideration is the etch uniformity as defined by Equation (1). A uniform etch would be one where the etch rate is equal across the wafer. Most RIE etches exhibit different etch rates across the surface of the substrate since no process is perfectly uniform. Often single substrate RIE processes exhibit a so-called “bulls eye” pattern on the surface were there are concentric rings of constant uniformity that vary from the center to the edge.

(1) $$\mathrm{Uniformity}\;\left(10\%\right)=\frac{\mathrm{Max}\;\mathrm{Etch}\;\mathrm{Rate}-\mathrm{Min}\;\mathrm{Etch}\;\mathrm{Rate}}{\mathrm{Max}\;\mathrm{Etch}\;\mathrm{Rate}+\mathrm{Min}\;\mathrm{Etch}\;\mathrm{Rate}}\times \left(100\%\right)$$

The mask selectivity is also an important and is defined as the etch rate of the material relative to the etch rate of the masking layer. A level of higher mask selectivity is more attractive since this allows the mask to be made from a thinner material layer, and a thinner mask improves the dimensional control of the features being etched in the material layer.

The amount of lateral etch is an important consideration and is defined as the anisotropy as given in Equation (2). This is the etch rate of that material that is etched laterally resulting in undercutting of the masking layer relative to the vertical etch rate. It is desired that there is no lateral etching. However, some amount of lateral etching is unavoidable.

(2) $$\mathrm{Etch}\;\mathrm{Anisotropy}=1-\frac{\mathrm{Lateral}\;\mathrm{Etch}\;\mathrm{Rate}}{\mathrm{Vertical}\;\mathrm{Etch}\;\mathrm{Rate}}$$

Ideally, the lateral etch would be zero, with a resultant anisotropy of 1. In practice, this is not the case.

The aspect ratio of an etch is another important parameter and is given by the ratio of the depth of the etched feature to its width. Higher aspect ratios are more challenging since as the etched feature gets deeper for a given width, the diffusion of reactive species and by-products of the etch from the bottom of the features becomes increasingly difficult due to transport limitation effects.

The loading effect is defined as an etching rate that is dependent on the amount of the substrate area being etched. Specifically, the etching of larger amounts of the substrate surface results in a decreased rate of etching relative to smaller amount of substrate area being etched. Loading effects can be at both a microscale level (e.g., at an individual feature or die) and a macroscale level (e.g., across the substrate). Loading effects are caused by the depletion of reactants at the etched features. It can be mitigated by reducing the rate of etching or increasing the available reactants for etching.

Etch loading can be detrimental on the resultant etch uniformity. For example, it has been shown that if a large percentage of the substrate surface is exposed to etching, from approximately 8% exposed area to approximately 100% exposed area, that the etch rate can be reduced by more that 50% and the non-uniformity can be increased from 2% to 35% [16]. Therefore, as a general rule, it is prudent to keep the percentage of substrate area exposed for etching to below 10%.

Etch lag refers to an etch rate that is lower in smaller sized features than in larger ones. If the etched patterns have a large range of feature sizes across the wafer or die, the larger features will faster than the smaller ones. In order to complete the etch, the larger features will be over etched before the smaller features are completely cleared resulting in a non-uniformity. The over etch in the larger features can also result some lateral etching of the features and undercutting of the masking layer resulting in loss of dimensional control. The amount of etch lag in any situation is complicated and depends on the dimensions of the smallest features as well as the differences in the sizes of the features as well as the amount of area exposed to the etchant. A solution for etch lag is to make all of the etched features have the same smallest dimension across the substrate. It has also been shown that etch lag can be reduced by adjusting certain processing parameters [17].

A parameter sometimes used in describing the etch lag in RIE etching is called the aspect ratio dependent etching (ARDE). This refers to the fact that the etch rate depends on the aspect ratio of the features being etched. As the aspect ratio increases, wherein the ratio of the depth to the width of the feature increases, it becomes increasingly difficult for the reactants to diffuse to the bottom of the feature as well as reaction by-products to diffuse out into the gas stream to be pumped out of the chamber. The impact is to slow the etch rate.

Micro-trenching is an increased etch rate near the bottom of the sidewalls of the etched features resulting in essentially a groove around the perimeter of the features that is slightly deeper than the floor of the feature [18]. This is believed to be caused by scattering of the ions of the etch process from the sidewalls of the features.

When RIE etching has a dielectric material etch stop, such as an etch that is conducted though the device layer of a silicon-on-insulator (SOI) wafer, the silicon sidewalls at the floor of the features (and the interface with the buried oxide layer of the SOI wafer) may exhibit a lateral etch larger than the lateral etch along the upper portions of the sidewall [19]. This is termed “notching” and is believed to be due to the charging of the dielectric layer caused by the impinging charged species that results in an electrical field that can steer the trajectories of the plasma ions as they near the feature floor.

## 5. Inductively-Coupled Plasma (ICP) RIE Etching

While RIE has found widespread use in microfabrication, it has not been useful for deep, high-aspect-ratio etching of silicon since the depths of the etchants are generally limited to a few microns. Bulk micromachining requires etching depths of hundreds of microns, including completely through the substrate (i.e., 500 microns or more) as well as high-aspect ratios [13].

**Figure 5:**
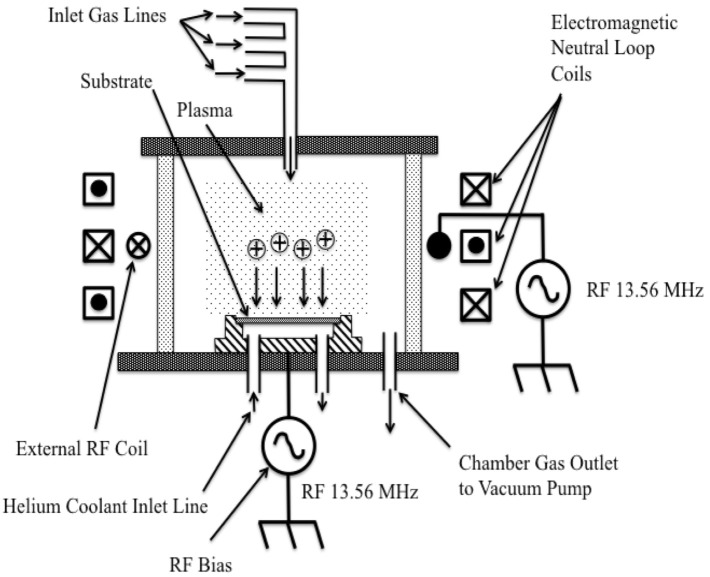
Illustration of an inductively-coupled plasma (ICP) reactive ion etch system configuration. In this system, there are two RF generators, one to create and sustain the plasma and the second to bias the reactants to the substrate [20].

Another form of RIE that is better suited for deeper and high-aspect-ratio etching is called the inductively-coupled plasma (ICP) etch system configuration. One version of an ICP etch system is shown in Figure 5. In the ICP etch system, the plasma is generated with an RF powered magnetic field and a separate RF generator directs an electrical field to steer the reactants toward the substrate and obtain a highly anisotropic etch result. The advantage of the ICP configuration is that very high plasma densities are created at low operating pressures thereby allowing a higher etch rate, while simultaneously obtaining a more anisotropic etch profile due to the biasing of the reactants via the bottom electrode that is de-coupled from the field sustaining the plasma.

## 6. Deep Reactive Ion Etching (DRIE) of Silicon

While ICP etching allows deeper etches into materials, it is not sufficient for deep, high-aspect-ratio anisotropic etching of single-crystal silicon. Fluorine-based plasma chemistries are well-suited for silicon etching due to the high chemical reactivity of fluorine radicals and silicon combined with the high volatility of the silicon fluoride reaction products [21]. However, these chemistries are so reactive and the reaction by-products are so volatile, that anisotropic etching of silicon can only be achieved by some means of passivation of the etched feature sidewalls [22]. In general, there are two primary mechanisms that sidewall passivation is obtained. One is a continuous sidewalls passivation and the second is a cyclical passivation [15].

In the 1990s, several new plasma etching technologies were developed that offered the possibility of etching tens or even hundreds of microns into single-crystal silicon [23,24,25,26,27]. This technology has been given the moniker of deep reactive ion etching (or DRIE) and was very quickly adopted by MEMS, NEMS, microsystems and micromachining developers as the bulk silicon micromachining method of choice. It enables implementation of very deep and very high-aspect-ratio etches to be performed into silicon substrates. The sidewalls of the etched holes are nearly vertical and the depths of the etches can be hundreds or more microns into the silicon substrate.

## 7. Cyrogenic DRIE

Cryogenic deep, high-aspect-ratio plasma etching uses fluorine radicals created from sulfur hexafluoride gas (SF_6_) in the plasma discharge as the etching species [25,26,27,28]. Passivation is achieved by injection of oxygen gas (O_2_) into the process chamber that forms oxygen radicals that react to oxidize the exposed silicon surfaces forming a thin-film layer of silicon oxide that protects the surfaces from attack by the fluorine radicals. Additionally, another process gas such as trifluoromethane (CHF_3_) may be added to allow the useable process window to be enlarged. Importantly, cryogenic etching is performed at temperatures of approximately 173 degrees-K by using liquid nitrogen to cool the substrate electrode. This lowers the amount of oxygen required to a few percent of the total gas flow in order to obtain passivation and anisotropic etching while also maintaining an acceptable etch rate and mask selectivity. The low process temperature significantly reduces the erosion of the passivation on the sidewalls that receive little to no ion bombardment. The bottoms of the features are bombarded with the full energy of the ions and this selectively removes the passivation at those locations thereby enabling the etch to proceed deeper into the substrate. The use of photoresist masking layers is difficult with cryogenic etching since the resist is a polymer and can crack with the low temperatures. However, some resists with special treatments may still be useable. Silicon dioxide hard masks are more commonly used. Typical etching rates for cryogenic etching are approximately 4 to 5 um per minute and the mask selectivity is approximately 100 to 1 for a silicon dioxide masking layer [27,28].

The main disadvantage of cryogenic etching is the low process temperature. Since the substrate is at such a low temperature, it attracts particulates and contaminates in the process chamber that can lodge onto the surface thereby creating micro-masking effects exhibited in the form of etching grass [15].

Cryogenic dry etching does have an advantage over cyclical DRIE since there is no scalloping of the sidewalls of the etched features (as explained below) and the sidewalls can be optically smooth. Therefore, cryogenic etching is primarily used for optical and photonic applications where sidewalls smoothness is often very important.

## 8. Bosch^TM^ DRIE

The cryogenic DRIE process for silicon etching uses continuous passivation, that is really only practical at very low temperatures. While this process allows deep and high-aspect-ratio etches to be performed in silicon, the process is prone to micro-masking effects [15]. This is due to the balancing of the formation of silicon oxides as passivation layers that require significant ion energies to remove and the need to remove these passivation layers at the trench floors of the features being etched. In addition to being prone to micro-masking, the higher ion energies also reduce mask selectivity.

Another DRIE process, called the Bosch^TM^ process, separates the passivation and etching into two different cycles. This enables the process gases for each cycle to be controlled independently [23]. Moreover, the passivation is performed using polytetrafluoroethylene (PTFE), or Teflon^TM^, that is not as hard as the silicon oxide passivation of cryogenic etching and therefore requires less ion energy to remove from the surfaces during the etch cycle.

**Figure 6:**
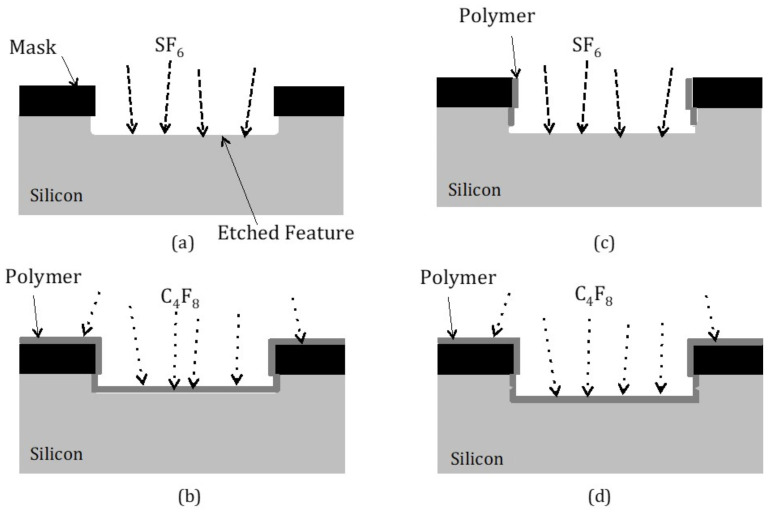
An illustration of the mechanism of the Bosch^TM^ process for the DRIE etching of silicon [13]. (**a**) SF6 is used to create the fluorine-based reactive species to etch the silicon. (**b**) the etch tool turns off the SF6 process gas and switches on the C4F8 process gas. (**c**) the process gas C4F8 is switched off, the etching process gas SF6 is turned back on. (**d**) the SF6 is turned off and the polymerization gas C4F8 is turned back on to again.

Figure 6 illustrates how the Bosch^TM^ deep reactive ion etching process is performed [13,15,23]. The etch is a cyclical dry plasma etch process that alternates between a high-density plasma to etch the silicon in one part of the cycle and then deposit an etch resistant polymer layer on the sidewalls in the other part of the cycle. The etching of the silicon is performed using a SF_6_ chemistry, whereas the deposition of an etch resistant passivation polymer layer on the sidewalls uses a C_4_F_8_ chemistry [23].

As shown in Figure 6a, in the first part of the first cycle process gas, SF_6_ is used to create the fluorine-based reactive species to etch the silicon. As discussed above, SF_6_ is a commonly used process gas for reactive ion etching of silicon. A mask of some SF_6_ resistant material, such as photoresist or silicon dioxide, has been patterned on the substrate surface to expose selected areas of the silicon substrate surface for etching. The etching into the exposed silicon is for a relatively short period of time (e.g., 1 s) and therefore precedes a limited depth into the silicon substrate surface. Then, as shown in Figure 6b, the etch tool turns off the SF_6_ process gas and switches on the C_4_F_8_ process gas resulting in the deposition of a relatively uniform coating of passivation polymer over the entire substrate surface. When the C_4_F_8_ process gas is turned off, that completes one full etch and passivation cycle.

After the process gas C_4_F_8_ is switched off, the etching process gas SF_6_ is turned back on as shown in Figure 6c. This results in the removal of the polymer layer that is directly exposed to the silicon etchant gas reactive species and plasma ions at the bottom surfaces of the etch trench. However, the polymer on the sidewalls is able to remain a longer period of time due to the fact that it is not being directly bombarded by the ions. The result is that the fluorine reactive species are able to etch some depth into the exposed silicon trench once the polymer passivation on the trench bottoms has been removed. The polymer passivation of the sidewalls prevents the sidewalls from being attacked and therefore prevents laterally etching of the silicon trench sidewalls from occurring. Then, as shown in Figure 6d, the SF_6_ is turned off and the polymerization gas C_4_F_8_ is turned back on to again resulting in the deposition of a thin polymer layer over the silicon substrate. This alternating cycling of the process gases continues repeatedly until the desired etch depth into the silicon substrate is obtained.

Mass flow controllers and automated valve mechanisms in the process tool are used to precisely control the cycle the etch chemistries flow rates during the etch cycles. The anisotropy of the etch deeply into silicon is based on the cyclical nature of this process and the fact that the polymer at the bottom of the etch pit is removed faster than the polymer from the sidewalls. The sidewalls of features made with DRIE etching are not perfectly (or optically) smooth and if the sidewall is magnified under SEM inspection, a characteristic scalloping pattern is seen in the sidewalls [13,15]. This is in contrast to the cryogenic DRIE process. However, the Bosch^TM^ DRIE process is far less susceptible to micro-masking effects [15]. The vast majority of DRIE silicon etch systems today employ the Bosch^TM^ process and there has been a number of reviews for the use of this type of DRIE in the literature [29,30,31,32,33,34].

DRIE silicon etch tools, whether cryogenic or the Bosch^TM^ process, are single wafer systems and consequently the etching rate is an important consideration. The etching rates on most of the early DRIE systems ranged from approximately 1 to 5 microns per minute. This etching rate made it difficult for DRIE to be used in production since the cost was considered too high for deep etches.

With subsequent generations of DRIE systems using the Bosch^TM^ process, the etching rate of silicon has been significantly improved with recent generations of the Bosch process etch systems reaching 20 to 25 microns or higher per minute. Recent versions of DRIE systems also have improved the gas switching characteristics by reducing the cycle times. This has resulted in a reduction in the scalloping effects on the trench sidewalls [13,35].

Photoresist and silicon dioxide are commonly used masking layer for DRIE etching using the Bosch^TM^ process. The typical selectivity of the etching silicon relative to the masking material layers composed of either photoresist or silicon oxide are approximately 75 to 1 and 150 to 1, respectively. For a through wafer etch, a relatively thick photoresist mask layer will be required. With process optimization to the features being etched, the aspect ratio of the etch can be as high as 50 to 1 [36,37], but in practice tends to be 15 to 1.

The process recipe for DRIE may need adjustment depending on the amount of exposed silicon due to loading effects in the system, with larger exposed areas etching as a much faster rate compared to smaller exposed areas (i.e., etch lag). Consequently, the etch should be characterized and optimized for the exact mask feature and depth to obtain good results [38,39,40,41,42,43]. It is advisable that microsystems device designs implemented using DRIE keep the width of the etched features at the same dimension across the entire wafer if possible.

**Figure 7:**
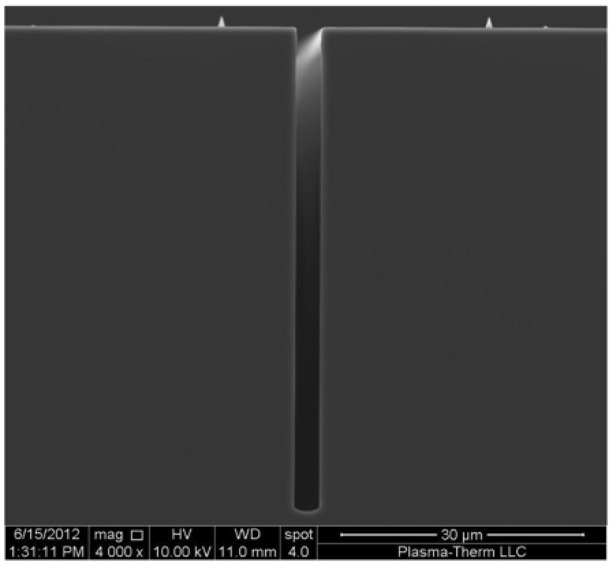
SEM of the cross section of a silicon wafer demonstrating high-aspect-ratio and deep trenches that can be fabricated using DRIE technology. This etch was performed using a PlasmaTherm Versaline DSE system [13].

Figure 7 is the cross section SEM of a deep, high-aspect-ratio silicon trench fabricated using the Bosch^TM^ process DRIE technology on a relatively new high etch rate tool. This etch was performed using a commercially available PlasmaTherm Versaline Deep Silicon Etch (DSE) etch system. This tool has etch rates as high as 20 microns/min, virtually no scalloping in the sidewalls, and minimal lateral etch (i.e., undercut). These characteristics are important to accurately controlling the dimensions of the etched features [13].

The achieved dimensional variations in DRIE depend on the etch depth, tool, recipes, mask design, loading effects, and other factors. The principal dimensional variations of interest are the uniformity of the etch depths across the substrates, and the lateral undercut of the etch mask. The across wafer uniformity of DRIE using the Bosch process has been reported to be approximately ±1% if the etched features have the same size on the masking layer [44].

The lateral etch rate depends on the aspect ratio and the depth of the etched features. As noted above, an aspect ratio of 15 to 1 is a typical value. Therefore, if a DRIE etch is performed that is L microns in depth, and assuming an aspect ratio of 15 to 1, this would mean that the lateral etch was equal to L/15 or 0.067 L. This lateral etch would be on both sides of an etched feature and therefore would be multiplied by 2 to give 0.134 L. As seen from this example, the lateral etch can be substantial [13]. It should be noted that the masking layer for the DRIE etch can be biased by the lateral etch amount to result in etched feature dimensions more closely aligned with the desired dimensions.

There are some general guidelines applicable to deep, high-aspect-ratio etching of silicon using the Bosch^TM^ DRIE process. As noted above, the etching rate tends to decrease with depth, especially for deep, high-aspect-ratio features. This is believed to be due to reduced ion bombardment being able to reach the bottom of the trenches. As a consequence, this effectively shifts the process to more passivation compared to earlier in the etching process [15]. Eventually, if the passivation completely overtakes the etching the etching will completely terminate.

A solution to this problem is to modify the etch recipe such that the amount of passivation is reduced [38,39]. This is most readily achieved by adjusting the ratio of the etch time to that of the passivation time in each cycle. This can be performed either in a stepwise fashion or continuously as the trench gets deeper. Another option would be to increase the etch time in each cycle while leaving the passivation time unchanged. The challenge is to find a suitable recipe that varies the ratio of etch to passivation and also can allow the etch to proceed while keeping the sidewalls vertically oriented and straight completely along the profile. The exact recipe for the ratio of etch to passivation times in the cycles as well as other etcher tool settings such as plasma power and bias power will need to be determined for each feature design and other process conditions. It should be noted that the process modifications described above for increasing the etch feature depth and aspect ratio assumes that all of the features have the same or nearly the same sizes.

Etch lag was defined above. It can be avoided by ensuring all the features to be etched have the same widths [13]. However, in some cases, the design requires features with significantly varying widths. In those situations, other solutions are needed [17,45]. Etch lag is believed to be mostly determined by the mean free paths of the species since this has a significant impact on their transport in narrow trenches. The most direct method for modifying the mean free path is to adjust the process pressures. Since the Bosch^TM^ DRIE process is composed of two parts wherein the pressures of each part can be individually controlled, the etch lag can be reduced by increasing the process pressure during the passivation step relative to the pressure during the etch step. Additionally, it has been found that decreasing the temperature of the substrate during processing from 40 °C to 0 °C can enable the etch to exhibit nearly lag free behavior over a range of aspect ratios [41]. A reduction in the notching effect can be achieved by pulsing the electrode that the substrate sites on to discharge the dielectric layer [42,43]. Techniques to reduce the scalloping on the sidewalls of the features using DRIE have also been recently reported [46].

DRIE etching has developed to the point where the etch rates are sufficient for production [35]. DRIE is now commonly used for bulk micromachining of single-crystal silicon for a wide range of applications—inertia sensors [47,48,49,50,51], pressure sensors [52,53,54], microphones [55], resonators [56], microfluidics [57,58,59], and others [60,61].

## 9. Deep, High-Aspect-Ratio RIE of Fused Silica, Quartz and Glass

Various forms of silicon dioxide materials including fused silica and quartz have a number of attractive material properties for applications in microsystems devices [62,63,64,65,66,67,68]. For example, fused silica exhibits: a high quality (Q) factor; high stiffness; chemical inertness; good thermal stability; small visco-elastic losses; low thermal expansion; good thermal shock resistance; low dielectric constant and low dielectric losses; excellent optical transparency ranging from deep ultraviolet to the far infrared; and low thermal conductivity. Additionally, quartz, a crystalline form of silicon dioxide is a piezoelectric material and is an excellent material choice for a number of sensor, actuator, and electronic applications. Likewise, amorphous silicon dioxide has many attractive properties for microsystems device applications such as low thermal conductivity, low electrical conductivity, transparency, good stability, and biocompatibility [69].

Despite the practicality of the various forms of silicon dioxide materials, the fabrication methods for bulk micromachining has been mostly constrained to macro-scale machining technologies such as crystal cutting, sawing, grinding, and wet etching techniques. As a result, the dimensional control of these fabrication methods is not good and these methods are not amenable to wafer fabrication methods.

While dry plasma etching of silicon dioxide has been available in IC production for a number of years, it has been mostly limited to depths of a few microns or less, very limited aspect ratios, and typically non-vertical sidewalls of the etched features [70]. Silicon dioxide is considered a difficult material to plasma etch because it is resistant to most reactive chemical species commonly used in RIE. The capability to make very deep (more than 10s of microns), small-dimensioned devices and device features with high aspect ratios and vertical etched sidewalls in these materials has not been available to microsystems developers until recently.

The ability to etch very deep and high-aspect features into fused silica, quartz and glasses using a reactive ion etching process has been recently reported [20,71,72,73,74,75]. Pedersen and Huff reported an extensive design of experiments (DOE) by varying the most important etch process parameters over a range of values, subsequently performing measurements on the etch samples, and then conducting statistical analysis on the resultant measurements to determine the optimal process tool settings (i.e., recipe) resulting in the desired outcome. Importantly, these experiments were conducted on a tool especially designed to etch hard to etch materials such as silicon oxides. These experiments are reported in detail elsewhere [20,75,76,77]. Etches over 200 microns deep, with the ability of etching completely through 500 microns or thicker fused silica substrates, with high-aspect-ratio features of over 10 to 1 and nearly vertical sidewalls in the etched features were demonstrated [20].

Deep high-aspect-ratio plasma etching of fused silica has been described elsewhere and can be summarized as follows [78,79,80,81,82]. the etch process employs a continuous polymerization-dissolution process based on the ionization of C_x_F_y_ fluorocarbons (possible source gasses include CF, CF_2_, C_2_F_3_, and C_2_F_4_); the polymerization deposited onto the silicon dioxide surfaces part dissolves the silicon dioxide in a complicated chemical interaction of fluorination, desorption and passivation. The dissolution of the silicon dioxide occurs as an interaction between the bombarding ions from the plasma and the solid fused silica interface, resulting in the removal of silicon dioxide.

The directionality of the bombarding ions results in a preferential removal of the polymer at the bottoms of the features to achieve anisotropy in the etched features. This etch process is similar to the behavior seen in silicon deep reactive ion etching (DRIE), with the important difference that the polymer in the fused silica etch process is formed continuously during the etch.

Introducing oxygen or argon gases into the plasma modifies the polymerization rates in the process. Specifically, oxygen ions chemically attack the polymer passivation while bombardment by argon ions physically sputters the polymer passivation. Modification of the etch rate, mask selectivity, and anisotropy can be obtained adding these gases into the process chamber during etching [20].

Since this etch process is a continuous process and not switched or cycled between two process chemistries such as in the DRIE Bosch process, there is no scalloping of the sidewalls of the etched features. Therefore, this etch is more similar to the cryogenic silicon DRIE etch. The etch rates of silicon dioxide are slower than DRIE of silicon, especially for deep, high-aspect-ratio features. Further, this etch process is somewhat prone to micro-masking. The micro-masking can be sufficiently severe to result in defects in the etched features, such as bridging defects across trenches. A nominal etch rate averaged over an etch having an aspect ratio of approximately 5 to 1, to a depth of approximately 215 microns had a rate of approximately 500 nm/min [20,76]. The mask selectivity using a nickel hard mask was approximately 10 to 1. A metal hard mask is required, particularly for deeper etches, since neither photoresist or oxide are sufficiently robust. Nearly vertical sidewall angles (e.g., 90 deg +/− 0.5deg) can be achieved. Additionally, the thickness of the mask is important to avoid faceting effects on the upper sidewalls of the etched features due to ion steering effects from metal hard masks was reported [75].

Commercially available tools for performing deep high-aspect-ratio etching of silicon dioxide include the ULVAC Technologies Neutral-Loop Discharge (NLD) 6000 etch system [83]. This is an inductively-coupled plasma (ICP) etch tool that has a unique feature of three separate, independently controlled, electromagnetic neutral coils positioned external and completely encircle the etch chamber. This allows the magnetic field shape and strength to modify the plasma and spatial redistribution of the ions, which in turn impacts the etch rate, the resulting sidewall angle, and etch uniformity. The magnetic neutral loop discharge configuration is used to simultaneously create a high-density plasma and low operating pressure (e.g., 10^11^ cm^−3^ at 10^−1^ Pa) to enable higher etch rates in hard to etch materials as well as enabling more directional, or anisotropic, etching to be performed [20].

**Figure 8:**
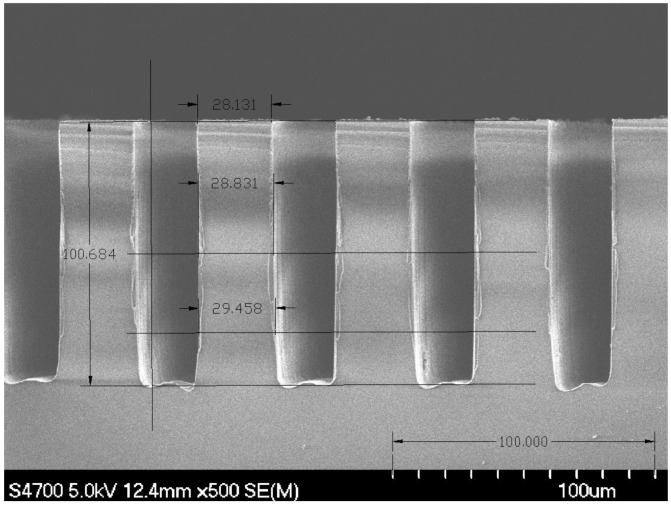
Scanning electron microscope (SEM) image of a cross section of fused silica sample after performing etch [20].

In order to limit micro-masking from re-deposited nickel in performing this etch, the etch cycle time was 30 min, with an oxygen (O_2_) clean cycle time of 30 s that is performed between each etch cycle [20,84]. Figure 8 shows a cross-sectioned substrate of fused silica after performing a deep high-aspect-ratio etch. The depth of this etch is between 100 and 125 microns and the aspect ratio shown in this figure is approximately 5 to 1. Deeper etches (up to 500 microns or more) or having higher aspect ratios (up to 10 to 1) are possible with this etch. The optimized recipe for performing deep etches into fused silica is as follows [20,84]:

RF Bias Power: 200 Watts
Substrate temperature: 15 °C
O_2_ gas flow: 9 sccm
Chamber pressure: 5 mTorr
C_3_F_8_ gas flow: 30 sccm
RF antenna power: 1950 Watts
Top magnet current: 6.1 Amps
Center magnet current: 10.2 Amps
Bottom magnet current: 6.1 Amps
Heat shield temperature: 150 °C
He cooling pressure: 5 Pascals.

A few additional points with regard to this etch process follow. First, although this etch process is prone to micro-masking effects that can be reduced using the cleaning cycles mentioned above, it has been found that the amount of micro-masking can be greatly reduced by limiting the amount of the substrate surface covered by the nickel hard mask to under 10% [84]. While this would appear to represent a large majority of the substrate being etched and therefore would be expected to cause loading effects, loading was not observed with this process. Additionally, little to no lag was seen as well. Second, the entire etch chamber should be thoroughly cleaned after each wafer etch. This includes scrubbing down the chamber walls using a mechanical abrasive. This is needed to remove a very thin layer of a polymer-nickel compound that builds up on the chamber walls and can fall off onto the substrate during etching. If these guidelines are followed then the number of micro-masking defects can be significantly reduced or eliminated.

The uniformity of this etch process was reported to be 2.41% in the lateral features across the substrate. The uniformity on the etch depths was 1.51% for an average etch depth of 132 microns [20,77].

## 10. Deep, High-Aspect-Ratio RIE of Silicon Carbide (SiC)

Silicon carbide (SiC) is a compound semiconductor material that has several very unique and desirable material properties including large energy band-gap; large mechanical stiffness; high electron and hole mobility; chemical resistance; extreme hardness; high mechanical quality factor (Q); high thermal stability; good thermal shock resistance; high thermal conductivity, electroluminescence; high abrasion resistance; and others [85]. As a result of these exceptional material properties, silicon carbide is an outstanding material choice for several important microsystems applications such as sensors for harsh environments, power and high temperature electronics, and others. Additionally, SiC substrates are often used for epitaxial growth of thin layers of single-crystal Gallium Nitride (GaN) semiconductor material for electronics and photonics. In some applications it is desirable to fabricate through substrate vias to increase the device density.

As a result of these unique material properties, silicon carbide is an excellent material choice for several Micro-Electro-Mechanical Systems (MEMS), micromechanical, and microelectronic applications in the commercial and defense sectors including MEMS sensors for harsh environments, power electronics, Milli-Meter-wave Integrated Circuits (MMICs), Light-Emitting Devices (LEDs), high-temperature electronics, and several others [86,87,88,89,90]. Additionally, SiC substrates are often used for epitaxial growth of thin layers of single-crystal Gallium Nitride (GaN) semiconductor material [91] wherein in some applications it is desirable to fabricate through substrate vias to increase the device density.

Many of the important microsystems device applications that require silicon carbide substrates need methods for bulk micromachining of this material. That is, they need methods to fabricate deep, high-aspect-ratio features into silicon carbide substrates. However, due to its chemical resistance, silicon carbide is an extremely challenging material to etch using any technique, particularly using a dry plasma etching processes.

Evan and Beheim reported using reactive ion etch processes to fabricate features in SiC [92,93]. However, the etch depths were reportedly restricted to ten’s of microns. Furthermore, they reported that the low etch rate, poor mask selectivity, numerous observed etch defects, particularly pillar formation and micro-trenching, in the etched features prevented obtaining high-fidelity, deep and high-aspect-ratio etching.

Okamoto et al., reported using reactive ion etching to fabricate through-substrate electrical connections in SiC, which is viewed as a key capability for implementation of GaN HEMT MMIC high-power amplifiers [94]. A high etch rate of approximately 2 microns/minute was reported. However, the etched features exhibited rough sidewalls, a severe RIE lag was observed, and the uniformity of the etch was quite large (+/− 4.1% uniformity). It is important to point out that nearly all of the work reported to date performed etching on SiC using the STS ICP DRIE silicon etcher that is commonly employed for DRIE etching of silicon.

The mechanisms of RIE etching of SiC using fluorinated gases have been reported by Pan et al., and Sugiura et al. [95,96] and highlight some of the major challenges in etching this material. The etching process can be summarized as follows. Silicon carbide exposed to fluorinated gases has a low rate of reaction and a very low rate of etch unless sufficient energy is supplied to the material by ion bombardment from the plasma. Specifically, the etching of silicon using fluorinated gases is much higher than carbon, leading to a carbon-rich surface as has been confirmed using auger electron spectroscopy (AES). Further, the surface of SiC subjected to ion bombardment during RIE etching also results in a carbon-rich surface since the sputtering yield of silicon is higher than that of carbon, which has also been confirmed using AES. Therefore, the rate-limiting reactions involve the carbon atoms and the ion bombardment of the surface during etching plays a critical role in the etch process, particularly in increasing the etch rate by breaking Si-C bonds thereby allowing reactions with fluorine to occur. The low reactivity of SiC to fluorinated gases requires high ion energies and this results in other issues that results in other challenges in etching SiC including poor mask selectivity, and a propensity to form deposits on the surface areas etched that can result in micro-masking. Even at high ion energies, the etch rate of SiC is generally low. The high ion energies require the use of a hard masking material layer for deeper etches. Moreover, even metal hard masks exhibit low mask selectivity [93]. Therefore, deep etches into SiC typically require relatively thick hard masks. Furthermore, the highly energetic ions can result in chemical species from the hard mask re-deposition onto the etched surfaces. This re-deposition combined with the inherently anisotropic nature of the etch can result in the growth of pillar defects in the etched areas.

**Figure 9:**
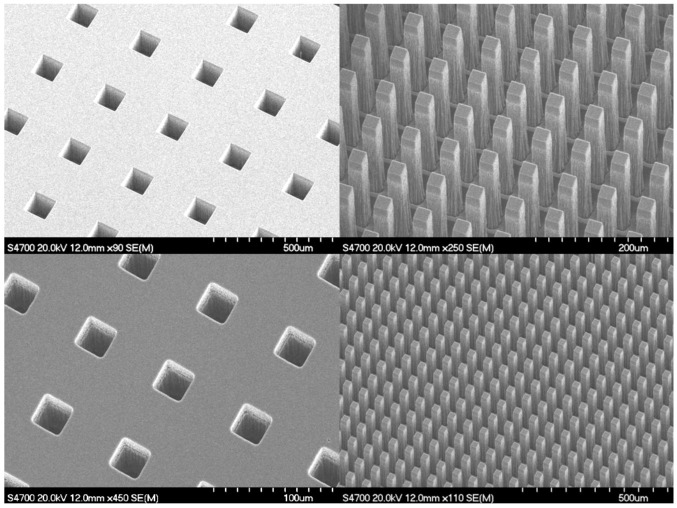
Scanning electron microscope (SEM) images of array of vias and posts after performing plasma etch using ULVAC NLD-6000. A 50 um via array (**top left**), a 25 um post array (**top right**), a 25 um via array (**bottom left**), and a 25 um post array (**bottom right**) are shown [97].

A deep and high-aspect-ratio etching capability for bulk micromachining of silicon carbide was recently reported [97,98,99]. This process was conducted on an inductively-coupled plasma (ICP) reactive ion etching (RIE) tool, specifically the ULVAC NLD-6000 etch tool [83]. This etch process, capable of performing etches of over 150 microns deep to etching completely through 500 microns or thicker silicon carbide material layers or substrates was demonstrated. A SEM image of the cross section of an etched SiC substrate is shown in Figure 9.

As can be seen, the recipe provides an etch that is deep and high in aspect ratio. The depth of the etched features was nearly 160 microns, with an average etch rate of approximately 1 micron/minute. Measurement of the sidewall angles indicated that they were 90 deg +/− 2.5 deg or nearly vertical. Further, the mask etch selectivity was measured to be approximately 130:1 using hard masks composed of 1 μm thick thin-film layers of copper (Cu) deposited using evaporation that was subsequently patterned using ion-beam milling [100]. The aspect ratio of the etched features was measured to be 12 to 1. No etch defects, such as pillars, were exhibited in the etched samples. The recipe for performing deep etches into silicon carbide is given below and the details of the experiments to develop this process can be found in the literature [97,98,99].

RF Bias Power: 100 Watts,
Substrate temperature: 12 °C,
O_2_ gas flow: 10 sccm,
Chamber pressure: 5 mTorr,
SF_6_ gas flow: 100 sccm,
RF antenna power: 2000 Watts,
Top magnet current: 6.1 Amps,
Center magnet current: 10.2 Amps,
Bottom magnet current: 6.1 Amps,
Heat shield temperature: 150 °C, and
He cooling pressure: 5 Pascals.

## 11. Deep, High-Aspect-Ratio RIE of Compound Semiconductors

Most III-V compound semiconductors can be RIE etched using chlorine or bromine process gases [15,101] as reported in a number of papers [102,103,104,105,106,107,108,109,110,111]. However, most of these materials pose several significant challenges for RIE etching. First, many of these compounds are composed of an element that is very reactive and volatile, such as arsenic or phosphorus, compared to the other element that is more resistant to etching or does not have volatile reaction by-products such as gallium and indium. Second, these materials are unusually prone to surface damage effects from etching that can significantly degrade the device performance. Third, many compound semiconductors are used for photonic applications where smooth etched surfaces are required.

High-aspect-ratio trench etches were reported in gallium arsenide (GaAs) using an ICP RIE etcher and Cl_2_ process gas [112]. To avoid roughness on the sidewalls as well as the formation of etch grass on the bottom of the trenches, the etch must be performed at very low pressures, typically approximately 0.2 Pa. The plasma source power was reported as 150 W, with a DC bias of 500 V in order to provide sufficient ion bombardment energy for equirate etching behavior. The aspect ratios of the etches was better than 5 to 1. The addition of Ar gas to the plasma improved the smoothness of the trench sidewalls [113]. Photoresist or oxide can be used as a masking layer. Metals can be used, but the grain structure of the metal can propagate into the GaAs features.

For GaAs/AlGaAs heterogeneous material layers it is sometimes desired that there be little to no etch selectivity between the two materials while in other situations high selectivity is preferable. It has been shown that using SiCl_4_ and SF_4_ process chemistry gases can provide a high selectivity of GaAs with respect to the AlGaAs [114].

**Figure 10:**
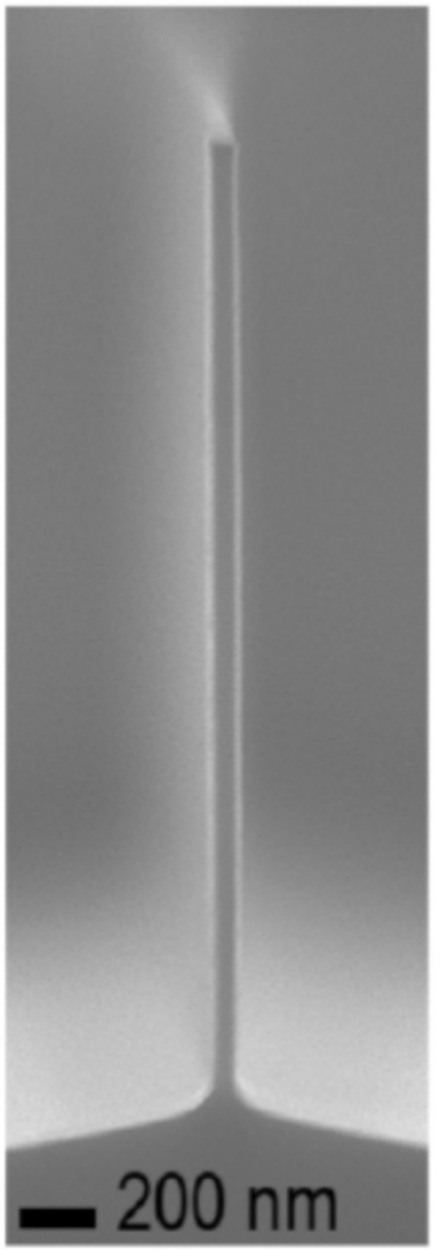
A GaAs nanowaveguide structure after passivation layer has been removed with an aspect ratio of >32, with a N_2_ flux of 11.8% ([115], used with permission from the Copyright Center and the contact author).

More recently, researchers reported the ability to etch nano-waveguides in GaAs and GaAs/AlGaAs heterogeneous material layers using a chlorine process etch gas in an ICP reactor with aspect ratios of over 30 as shown in Figure 10 [115]. A hard mask of a thin layer of PECVD silicon dioxide and chrome was used that was patterned using e-beam lithography and etched using a chorine plasma etch. Cl_2_ was used in combination with BCl_3_ and argon as the process gases. Cl_2_ is a known anisotropic etchant for GaAs and the addition of BCl_3_ aids with the etching of AlGaAs. The addition of argon gas helps in increasing the etch rate and provides for greater anisotropy. It was also found that adding N_2_ gas to the process helps in achieving higher aspect ratios. Chemical analysis (using EDX) indicated that the sidewall passivation was mainly composed of silicon oxide. There was little etch selectivity between GaAs and AlGaAs material types.

Deep high-aspect-ratio etching of indium phosphide (InP) has been demonstrated using Cl_2_ with argon using an electron cyclotron resonance (ECR) etching system at 500 W power and 100 W substrate power at 0.26 Pa pressure and a temperature of 20 °C [116].

High-aspect-ratio etching using an ICP etching system and HBr process gas has been demonstrated on InP. The aspect ratios varied from 20 to 40. The temperature of the substrate was 165 C. This high temperature is needed for the In reaction by-products to be sufficiently volatile. A 20 nm etched surface roughness was reported using this etch process [117].

## 12. Deep, High-Aspect-Ratio Etching of Piezoelectric Materials

The etching of piezoelectric materials is believed to be mostly by physical sputtering combined with some assistance from chemically reactive species in the plasma [118]. In fact, many of the reported reactive ion etching recipes for piezoelectric materials use inert gases to increase the ion densities in the plasmas to result in higher sputtering yields. As a result, many of the etched features have rough sidewall profiles. Aluminum nitride (AlN) has been successfully etched using chlorine-based chemistries [119,120] and etch rates of 0.23 microns per minute have been reported [121]. RIE etching of Zinc Oxide (ZnO) has been also reported with etch rate of 0.12 microns per minute using CF_4_/Ar, Cl_2_/Ar, and BCl_3_/Ar process gases [122]. Deep high-aspect-ratio reactive ion etching for lead zirconate titanate (PZT) has been reported by several researchers [123,124,125]. For example, high-aspect-ratio ICP etching of PZT was demonstrated with aspect ratios of approximately 5 to 1. A nickel on chrome/gold hard mask was used, with a mask selectivity of approximately 25 to 1. A process gas chemistry composed of SF_6_ and argon was used [125].

In comparison to semiconductor materials, there is a relatively small amount of reported research on deep, high-aspect-ratio RIE etching of piezoelectrics. Therefore, these materials represent a potential opportunity for further research.

## 13. Summary

This paper has reviewed the important developments in reactive ion etching to enable the implementation of deep and high-aspect-ratio features into various types of important semiconductor material substrates including single-crystal silicon; fused silica, glass and quartz; single-crystal silicon carbide (SiC); single-crystal gallium arsenide (GaAs); heterogeneous single-crystal gallium arsenide (GaAs) and aluminum gallium arsenide (AlGaAs); indium phosphide (InP); and piezoelectric lead zirconate titanate (PZT). Many of these technologies are critical for bulk micromachining applications as well as advanced forms of integrated circuits and 3-D integration. The processes for deep high-aspect-ratio reactive ion etching are dependent on a number of other technological developments including mechanisms for passivation of the sidewalls of the features during the etch or in one-half of the etch cycle; complex process gas chemical recipes that involve a number of constituents; and inductively coupled plasma etching systems that have high-density plasmas operating at low pressures. Many of these technological developments have taken several years in continuous development before they became available. Nevertheless, it is expected that significant future developments in further developing these technologies will continue since there is a considerable amount of active work in expanding the knowledge and understanding of plasma etching processes.
